# Bilateral dual iliac screws in spinal deformity correction surgery

**DOI:** 10.1186/s13018-018-0969-9

**Published:** 2018-10-19

**Authors:** Shigeto Ebata, Tetsuro Ohba, Hiroki Oba, Hirotaka Haro

**Affiliations:** 1 Department of Orthopaedic Surgery, Graduate School of Medicine, University of Yamanashi, 1110 Shimokato, Chuo, Yamanashi, 409-3898 Japan; 2 Department of Orthopaedic Surgery, Shinshu University School of Medicine, Nagano, Japan

**Keywords:** Bilateral dual iliac screws, Adult spinal deformity, S1 pedicle screw, Offset connector, Screw loosening, Pelvic anchor

## Abstract

**Background:**

Surgery for adult spinal deformity requires optimal patient-specific spino-pelvic-lower extremity alignment. Distal fixation in thoracolumbar spinal deformity surgery is crucial when arthrodesis to the sacrum is indicated. Although we had performed sacro-pelvic fixation with bilateral S1 and bilateral single iliac screws previously, iliac screw loosening and/or S1 screw loosening occurred frequently. So, the authors attempted to fuse spino-pelvic lesions with the dual iliac screws and S1 pedicle screws.

**Methods:**

Twenty-seven consecutive adult spinal deformity patients underwent thoracolumbar-pelvic correction surgery with bilateral double iliac screws between May 2014 and September 2015. Sagittal vertical axis, lumbar lordosis, pelvic tilt, sacral slope, T1 pelvic angle, and global tilt were assessed radiographically and by computed tomography both preoperatively and 24 months postoperatively. Iliac screw loosening, S1 pedicle screw loosening, and screw penetration of the ilium were evaluated 2 years postoperatively.

**Results:**

Only two patients (7.4%) at 1 year and three patients (11.1%) at 2 years presented with iliac screw loosening postoperatively. Loosening of the S1 screw occurred in three cases (11.1%) 2 years postoperatively. Displacement of the iliac screw occurred in eight cases (25%). Internal and external perforation of the ilium by the iliac screw occurred in six (22.2%) and three (11.1%) cases respectively. One reoperation was performed due to back-out of the iliac screw and rod breakage.

**Conclusion:**

Bilateral dual iliac screws and an S1 pedicle screw system achieve longer stability for spinal and pelvic fusion in adult spinal deformity patients, with few severe complications.

## Background

The pathology of adult spinal deformity (ASD) includes various components such as malalignment of the sagittal plane, lower angle of lumbar lordosis, and pelvic retroversion [1, 2]. The primary aim of surgery for ASD is to restore optimal patient-specific spino-pelvic-lower extremity alignment. Preservation of the lower lumbar and lumbar-sacral motion segments often results in implant failures, characterized by loosening or pullout of the pedicle screws, rod breakage, pseudarthrosis, or neurological deficits [3, 4]. Therefore, extension of spine fusion to the sacrum represents a significant improvement of clinical outcomes and decreases major complications [5]. Spino-pelvic fixation, stability, and correction of deformity have been reported with bilateral placement of single iliac screws [6, 7]. Although a combination of this procedure was effective in protecting the sacral screws from failure and sacroiliac joint degeneration, sacro-pelvic fixation with bilateral S1 and bilateral single iliac screws for ASD was associated with breakage or back-out of iliac screws, screw loosening, rod breakage, or pseudarthrosis of L5–S1 within 5 years postoperatively [8]. To overcome these complications, S2 alar iliac pelvic fixation has been developed and demonstrates better correction of pelvic obliquity with fewer complications [2]. This technique should be performed under fluoroscopic guidance to place screws bilaterally, although it involves exposing patients and surgeons to radiation. Screw penetration of the iliac table and articular violation can occur. Previously, dual iliac screw fixation or a double-rod double iliac screw method were reported to provide rigid fixation in spino-pelvic reconstructions associated with destructive metastatic lesions at the lumbosacral junction, or in a case with a sacral tumor requiring total sacrectomy [9]. Therefore, we have applied this method for ASD surgery using bilateral dual iliac screws as anchors in the ilium, and S1 screws to stabilize spino-pelvic fixation. The purpose of this study is to demonstrate the technique employing dual iliac screws and S1 pedicle screw fixation to fuse spino-pelvic lesions in ASD patients.

## Materials and methods

### Patients

All patients were considered candidates for thoracolumbar correction if fusion was indicated because of ASD and if a full course of conservative care had been exhausted. The inclusion criteria were symptoms including postural imbalance (leaning forward when walking and standing), low back pain, and/or gastro esophageal reflux disease, and a radiographic diagnosis of ASD defined by at least one of the following parameters: a coronal Cobb angle > 30°; a C7 sagittal vertical axis (SVA), which is the distance between the C7 plumb line and the posterosuperior edge of S1, > 5 cm; and/or pelvic tilt (PT), which is the orientation of the pelvis with respect to the femurs and the rest of the body, > 30°. Patients were excluded if they had rigid severe kyphosis, ankylosing spondylitis, a rounded back because of Parkinson’s disease, or if they had not been followed up for at least 1 year. Twenty-seven ASD patients were consecutively enrolled and underwent surgery between May 2014 and September 2015. Iliac screw loosening, pedicle screw loosening of S1, and misplacement causing screw penetration of the iliac table were assessed using computed tomography (CT) 1 and 2 years postoperatively. Screw loosening was defined as a lucent zone around the screw. Concurrence of at least two of the observers was mandatory to diagnose screw loosening [10]. The accuracy of screw placement was evaluated using criteria published by Neo et al. [11].

In addition, posterior-anterior and lateral radiographs of the entire spine in the standing position were investigated preoperatively, 12 months postoperatively. The SVA, lumbar lordosis (LL), PT, sacral slope (SS), T1 pelvic angle (TPA), and global tile (GT) were measured. All radiological analysis was performed two times by three independent doctors in a blinded fashion, and the measured values were averaged. This study was approved by our institutional review board (No. 1101).

### Surgical procedure

#### Overview

An anterior approach was selected to perform lateral interbody fusion (LIF) or posterior lumbar interbody fusion (PLIF) from the L1–2 or L2–3 to the L4–5 disc level to obtain adequate coronal and sagittal spine alignment in the ASD patients. Then, the patient position was changed to the prone position. The Ponte osteotomy and PLIF at the L5–S1 disc level were performed, and spinal kyphosis was corrected through cantilever force using bilateral S1 screws and bilateral dual iliac screws. In the case of loss in flexibility of spinal motion, a Ponte osteotomy, pedicle subtraction osteotomy, or vertebral column osteotomy was added.

#### Approach

**Fig. 1:**
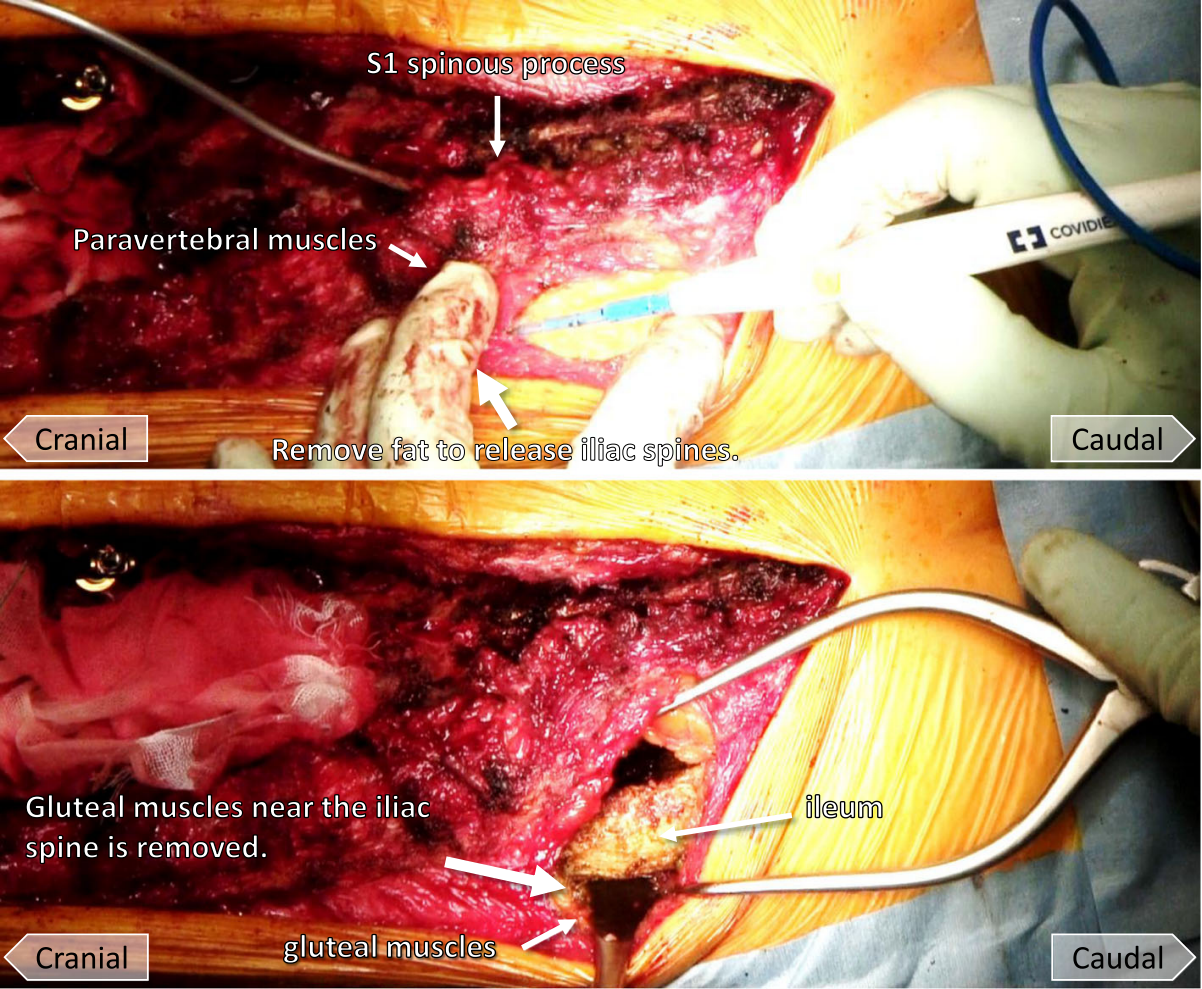
Photographs demonstrating the dual iliac screw technique. Muscle fascia on the ilium is incised to allow access for placement of the iliac screw (top). The Ilium between the posterior superior and inferior iliac spines is excised from the gluteal muscles (bottom)

**Fig. 2:**
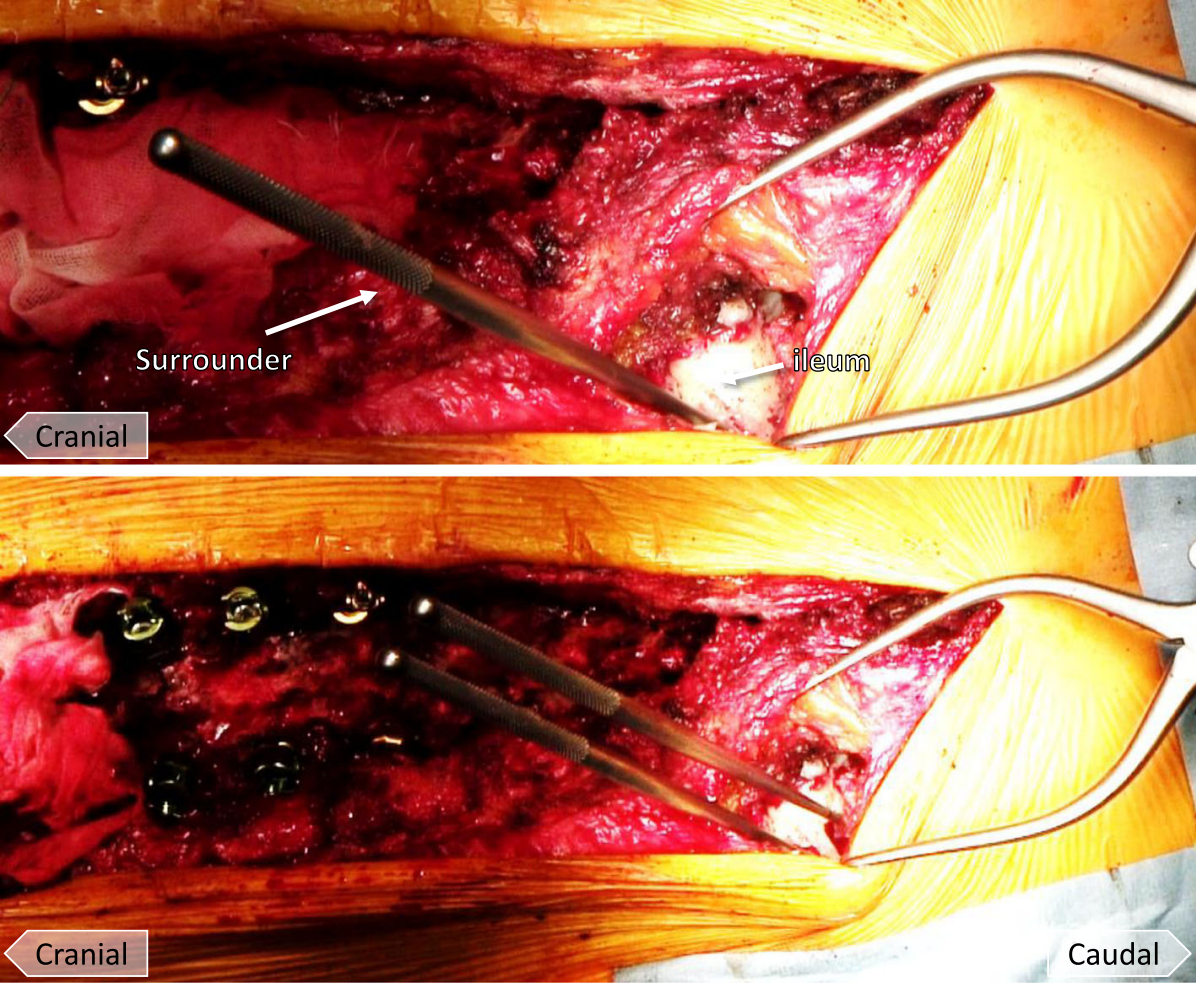
Photographs demonstrating the dual iliac screw technique. The surrounder is directed to the line between the anterior superior iliac spine and the greater trochanter (top). Another surrounder is placed as close as possible parallel to the primary surrounder direction (bottom)

**Fig. 3:**
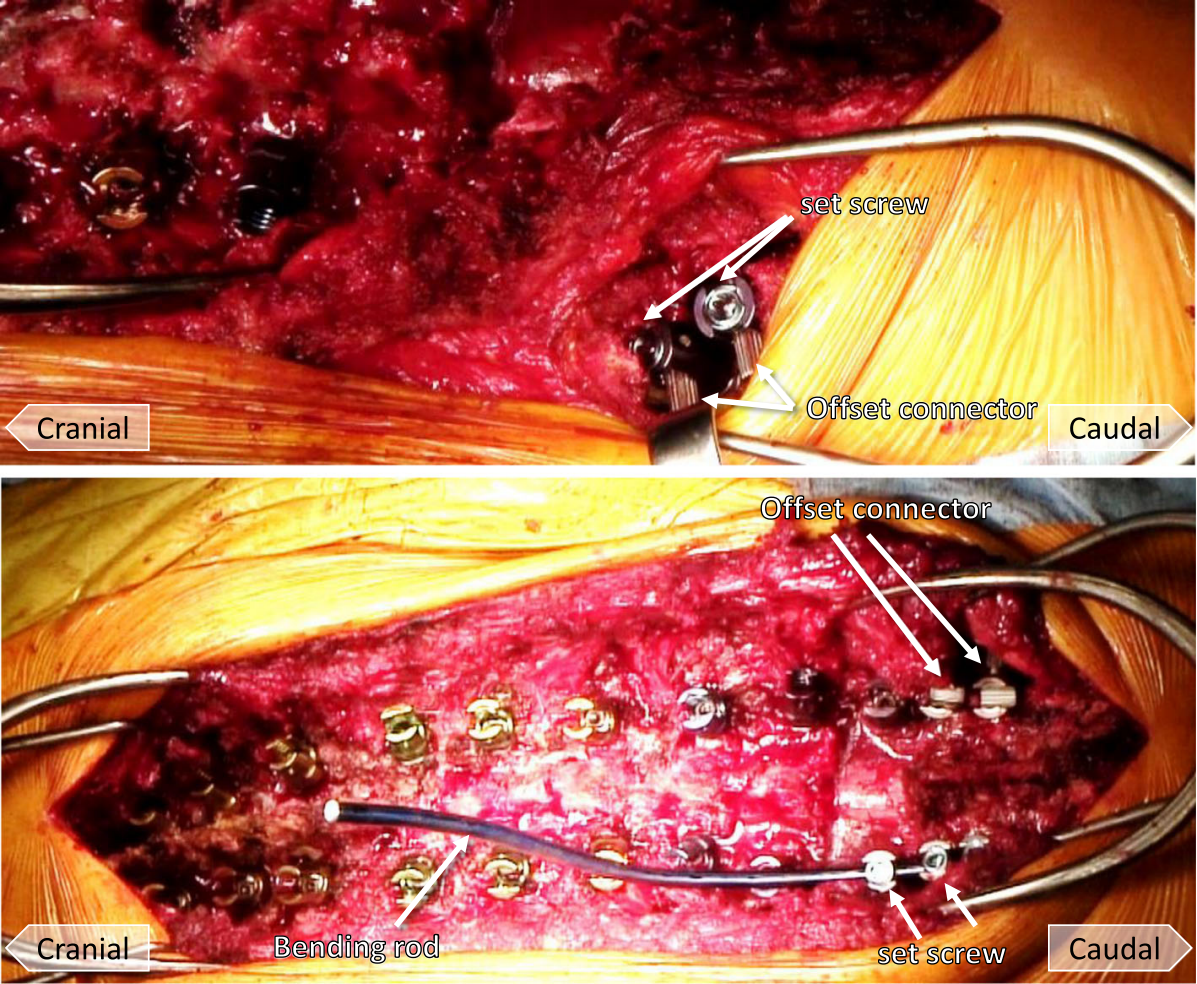
Photographs demonstrating the dual iliac screw technique. Iliac screws and the rod of the pedicle screws are connected with an offset connector (top). Lateral connectors bind the iliac screws to a rod which is concatenated with the S1 pedicle screw (bottom)

**Fig. 4:**
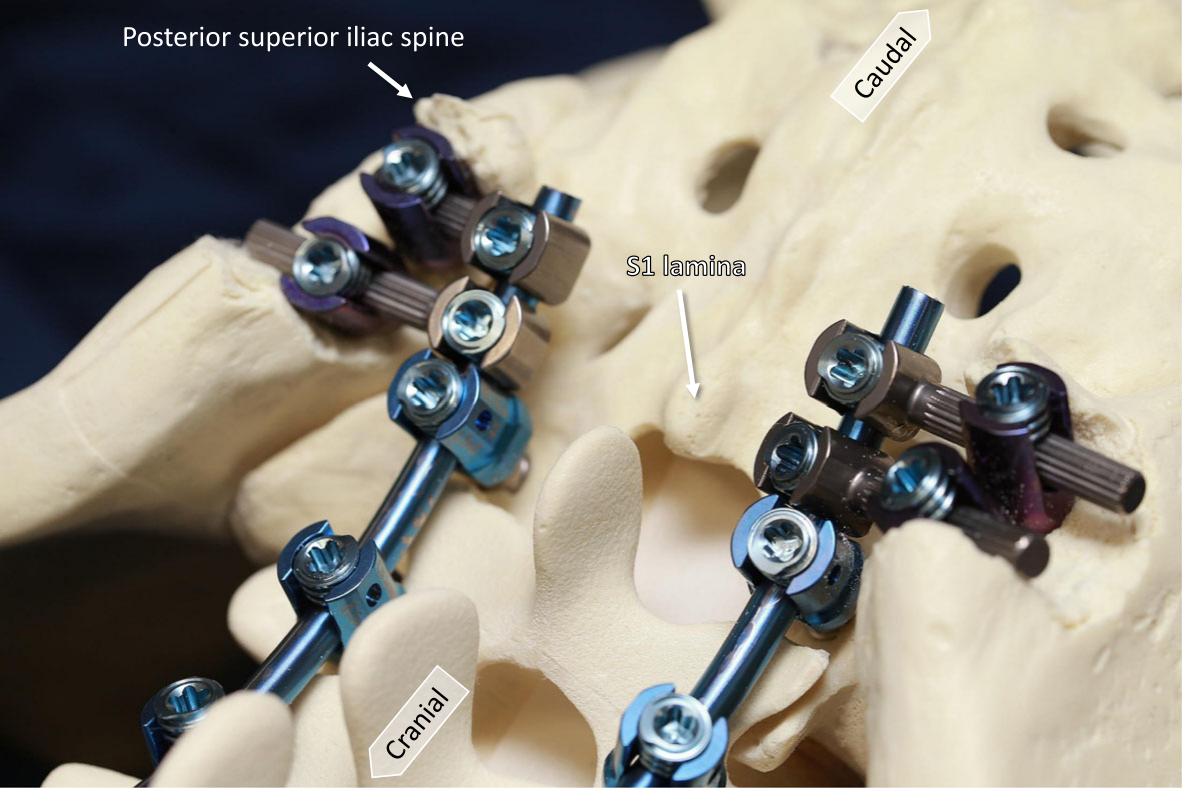
Photographs demonstrating the bone model. Dual iliac screws are bound to the S1 pedicle screw with a rod on each side, resulting in three rigid anchors on the right and left sides of the pelvis. The size of iliac screw commonly used is 7.5 mm (width) by 70 mm (length)

To place the screws, the paravertebral muscles including the multifidus muscles around S1 and S2 spinous processes first were removed to visualize the lamina and spinous processes. Muscle fascia on the ilium was incised to provide access to the iliac screws (Fig. 1). The posterior superior and inferior iliac spines were partially excised from the gluteal muscles to provide space to insert the dual iliac screws. A hole was made inside the paravertebral muscles from the ilium to the sacrum (Fig. 1). Cortical bone near the iliac spine was removed to the lamina of the sacrum using forceps to prevent skin failure of screw heads. Dual iliac screws were set in parallel without radiation exposure with a C-arm (Fig. 2). The dual iliac screws were bound to the S1 pedicle screw with a rod on each side (Fig. 3), resulting in three rigid anchors on the right and left sides of the pelvis (Fig. 4). Bone wax was spread on the ilium to avoid bleeding from bone.

#### Iliac screw setting

The size of the iliac resection must be large enough to set the dual iliac screws so that the heads of the screws are not prominent. The surrounder was directed to the line from the anterior superior iliac spine to the greater trochanter in a manner similar to the ball-tip method (Fig. 2) [12]. Another surrounder was punctured as close as possible to be parallel to the primary surrounder direction (Fig. 2). A bone hole was enlarged with a probe, and a screw of 7.5 mm in diameter and 70 mm in length usually was placed, although screws 7.5 to 8.5 mm in diameter and 50 to 80 mm in length were sometimes used.

#### Connection of iliac screws with the S1 pedicle screw

The length between the iliac screws and the rod of the pedicle screw was measured. Then, the pedicle screws were connected to the rod of the iliac screws with an offset connector through a hole inside the paravertebral muscles from the ilium to the sacrum (Fig. 3).

#### Correction of spinal deformity

**Fig. 5:**
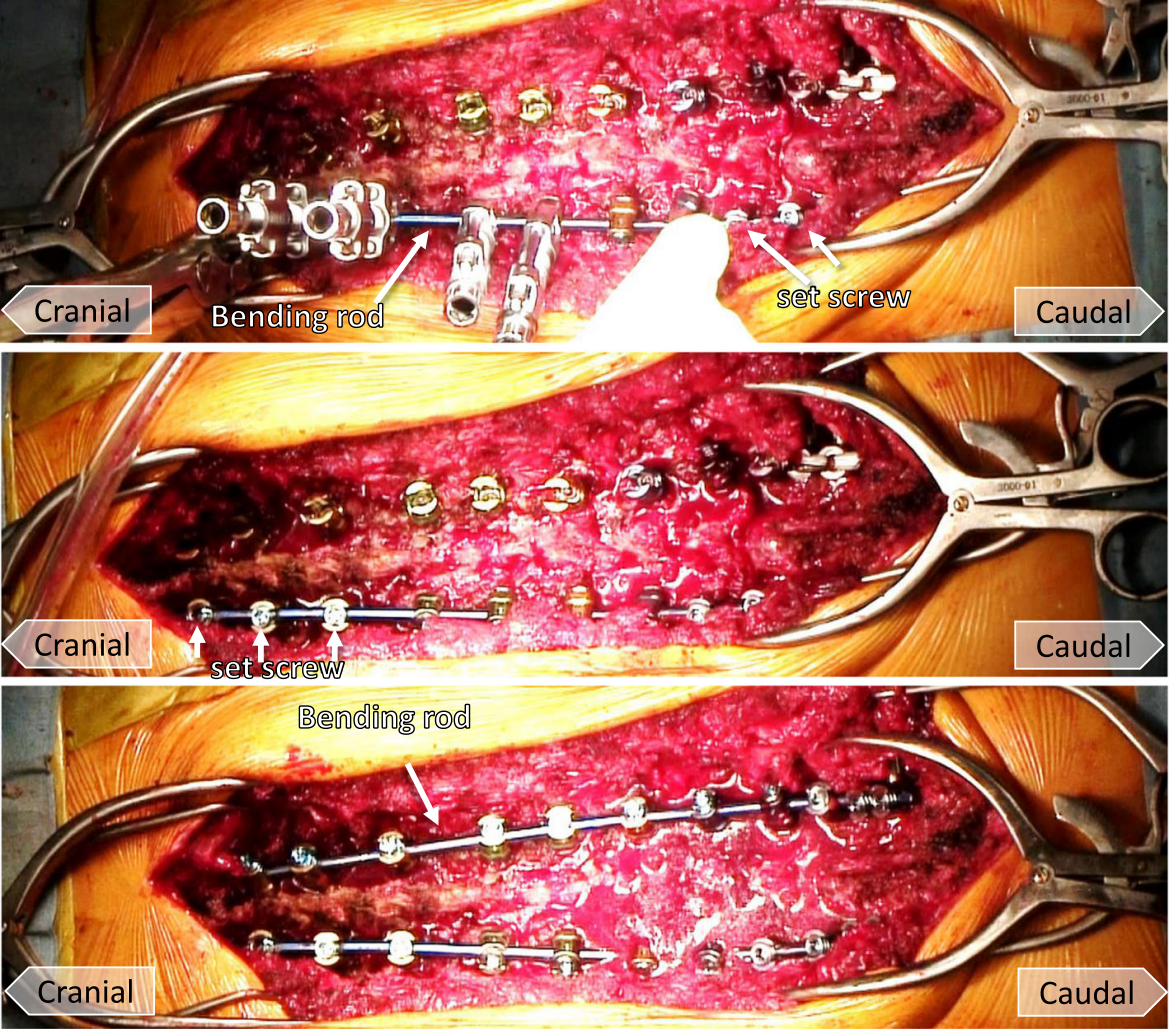
Photographs demonstrating the dual iliac screw technique. A rod is connected to each pedicle screw from caudal to cranial (top). It is important to disperse the correction force across each pedicle screw (middle). Another rod is set in the same manner on the opposite side (bottom)

Rods of adequate length extending from the cranial to caudal surgical level were prepared. The lateral connectors bind the iliac screws to a rod which was concatenated with the S1 pedicle screw (Fig. 3). The spino-pelvic deformity was corrected using a cantilever force technique with the pelvis retroverted, raising the pelvis to an optimal alignment. A rod connected each pedicle screw from caudal to cranial (Fig. 5). It is important not to impose a concentration of correction force on a specific screw but to disperse the force across the screws (Fig. 5). Favorable spinal alignment is usually obtained when the rod is set unilaterally. Another rod was set in the same manner on the opposite side (Fig. 5). Then, the rotation deformity of the vertebrae was corrected to the intervertebral disc space by compression-distraction force, and final fixation was performed.

#### Case presentation

**Fig. 6:**
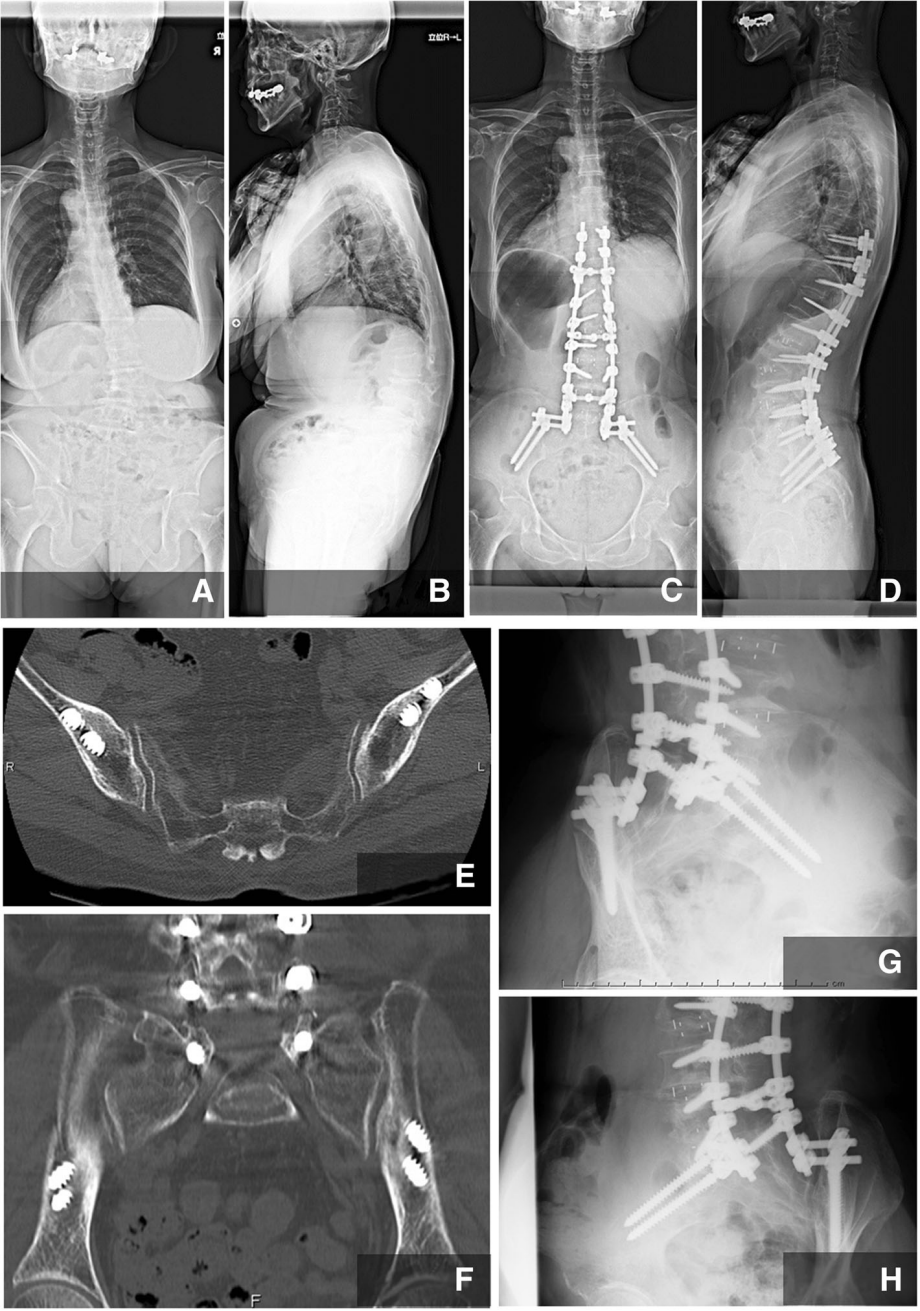
Case presentation. A 72-year-old female patient had adult spinal kyphosis and showed a postural imbalance, such as leaning forward or to the left side when walking and standing, resulting in claudication within 2 min. She underwent surgery including LIF at L2–3, 3–4, and 4–5 disc levels and posterior lumbar interbody fusion at L5-S1, and posterior corrective fusion from T10 to the ilium. **a** Preoperative A-P view, **b** preoperative lateral view, **c** postoperative A-P view, **d** postoperative lateral view, **e** postoperative CT axial view, **f** postoperative CT coronal view, **g**, **h** postoperative oblique views

A 72-year-old female patient had adult spinal kyphosis and showed a postural imbalance such as leaning forward or to the left side when walking and standing, resulting in claudication within 2 min. She underwent surgery including LIF at L2–3, 3–4, and 4–5 disc levels and posterior lumbar interbody fusion at L5–S1, and posterior corrective fusion from T10 to the ilium with bilateral S1 pedicle screws and bilateral dual iliac screws. It took 7 h and 18 min in surgical time and 179 ml of blood given intraoperatively (Fig. 6).

## Results

The patients’ age at surgery, gender, bone mineral density (BMD), number of osteoporotic vertebral fractures (OVF), operation time, bleeding, and location of upper instrumented vertebra are presented in Table 1. There are no previous surgical cases in this series.

**Table 1:** Preoperative patient characteristics

Variable	*N* = 27
Age at surgery (years)	69.6 ± 8.1
Female/male (*n*)	25/2
BMD (%YAM)	73.5 ± 10.7
Number of OVF	0.4 ± 0.7
Operation time (min)	487.9 ± 70.9
Bleeding (ml)	797.9 ± 508.7
Location of UIV (*n*)	
-Th7	5 (18.5%)
Th8–10	22 (81.5%)

Interval and ratio values represent the mean ± standard deviation

*BMD* bone mineral density, *OVF* osteoporotic vertebral fractures, *UIV* upper instrumented vertebra

Surgical complications presented by iliac screw loosening occurred in two cases 1 year and three cases 2 years postoperatively. Loosening of the S1 screw occurred in three cases. Displacement of the iliac screw occurred in eight cases 2 years postoperatively. Internal perforation of the ilium by the iliac screw occurred in six cases, whereas external perforation of the ilium by the iliac screw occurred 2 years postoperatively in three cases. One reoperation was performed due to back-out of the iliac screw and breakage of the rod.

### Radiographic outcomes

Surgery decreased the SVA from 86.7 to 14.9 mm and increased the LL from 15.1 to 52.4°. Surgery decreased the PT from 31.8 to 20.6° and increased the SS from 16.5 to 30.3°.

## Discussion

We have demonstrated how to perform dual iliac screw fixation as an iliac anchor to stabilize the spino-pelvic junction in ASD patients with osteoporosis. When considering a surgical strategy for ASD, the surgeon’s plan should consider not only global spinal balance but also pelvic alignment. Therefore, both global tilt and T1 pelvic angle are important to evaluate global alignment of the spine and the lower extremity through the pelvis. Proper alignment allows stability when standing [13, 14]. Pelvic fixation to stabilize the lumbosacral junction using L-rod instrumentation was reported as the Galveston technique in a case of spinal deformity [15]. This technique involved penetration of the L-rod from the ilium at the lower margin of the posterior superior iliac spine adjacent to the posterior surface of the sacrum. The Galveston rod technique was epoch making as a method of pelvic fixation, but resulted in frequent loosening or broken rods requiring reoperation [16]. As a substitute for that technique, bilateral single iliac screws with bilateral S1 screws were developed to provide a solid lumbosacral fixation and a solid foundation from sacrum to pelvis [8]. The authors observed no evidence of a long-term effect of the iliac screws predisposing the sacroiliac joints to degeneration. However, the head of the iliac screw is not in line with the cranial instrumentation and S1 pedicle screw. To address this, some complicated maneuvers must be performed, including removing paravertebral muscles near S1 and S2 and gluteal muscles from the ilium. In addition, it has been reported that a single iliac screw has a risk of loosening or pull-out when loaded cyclically, resulting in construct failure [17]. This postoperative complication is accompanied with pain and loss of instrumentation stability. Iliac screw loosening and/or S1 screw loosening occurred frequently in previous cases of ASD surgery.

Alternatively, S2-alar iliac pelvic fixation has been developed [2]. It is useful for this procedure to dissect paravertebral muscles to expose the surgical site and set the screw. This in-line approach allows for thoraco-lumbar spinal procedures and pelvic fixation. In addition, the screw is set deeper compared with the iliac screw through the skin. Screws longer than 100 mm can be used. Projection of screws on the ilium and violation to the articular cartilage of the sacroiliac joint have been reported [18]. Previous reports showed anomalies between the shape of the pelvis and the sacroiliac joint [5].

To settle these concerns, we have developed bilateral dual iliac screws and S1 pedicle screw fixation to strengthen the support of the pelvis. The initial idea for this procedure came from cases in which dual iliac screw fixation accompanied by total sacrectomy and tumor excision was used for lumbosacral tumors [9, 19]. The current procedure contains benefits, such as limited dissection of tissues around the ilium and sacrum, easy insertion of the iliac screw by a freehand ball tip technique without radiation exposure, and easy connection of iliac screws with a rod related to the S1 pedicle screw using an offset connector. We also avoid projection of the head of the iliac screw from the skin by resecting some of the ilium in an oval shape, because the prominence of the iliac screws was an indication for reoperation to remove them [8]. We have experienced only one patient complaint regarding a prominent screw head, resulting in reoperation. Our bilateral technique using three anchors in the pelvis gives rise to solid fixation and adequate spinal alignment after correction and distributes the force to each screw, reducing the load to each screw in ASD surgeries, L5-S disc level pseudarthrosis or L5 spinal tumor cases. In the current study, only 11.1% of patients from the dual iliac screw group presented with screw loosening more than 2 years postoperatively. A previous study with bilateral single iliac screws reported that iliac screw loosening occurred in 27.8% of patients and S1 screw loosening in 9.7% of patients 2 years postoperatively [20]. Based on that study and our results, the procedure using bilateral dual iliac screws may reduce surgery-related complications compared with the single dual iliac screw procedure.

However, this study has some limitations. First, it is a relatively small study with a limited number of patients. Second, the follow-up observation period of 1 year was short. Third, this study did not evaluate clinical outcomes. Fourth, this study did not compare surgical outcomes with bilateral dual iliac screws and a single iliac screw, or S2-alar iliac pelvic fixation. Future study is required to address these points.

## Conclusion

The bilateral dual iliac screw and S1 pedicle screw system provide 6 points as pelvic anchors and promise improved stability for long spinal and pelvic fusion for ASD. It is accompanied by few complications and may allow a superior correction over single iliac fixation to obtain ideal spino-pelvic alignment.

## Abbreviations

ASD: Adult spinal deformity
BMD: Bone mineral density
CT: Computed tomography
GT: Global tilt
LIF: Lateral interbody fusion
LL: Lumbar lordosis
OVF: Osteoporotic vertebral fractures
PLIF: Posterior lumbar interbody fusion
PT: Pelvic tilt
SS: Sacral slope
SVA: Sagittal vertical axis
TPA: T1 pelvic angle
UIV: Upper instrumented vertebra
